# Mitochondria-Targeted Antioxidant Therapeutics for Traumatic Brain Injury

**DOI:** 10.3390/antiox13030303

**Published:** 2024-02-29

**Authors:** Hiren R. Modi, Sudeep Musyaju, Meaghan Ratcliffe, Deborah A. Shear, Anke H. Scultetus, Jignesh D. Pandya

**Affiliations:** Brain Trauma Neuroprotection (BTN) Branch, Center for Military Psychiatry and Neuroscience, Walter Reed Army Institute of Research (WRAIR), 503 Robert Grant Avenue, Silver Spring, MD 20910, USA; hiren.r.modi.ctr@health.mil (H.R.M.); sudeep.musyaju.ctr@health.mil (S.M.); meaghan.l.ratcliffe.ctr@health.mil (M.R.); deborah.a.shear.civ@health.mil (D.A.S.); anke.h.scultetus2.civ@health.mil (A.H.S.)

**Keywords:** traumatic brain injury, mitochondria, free radicals, oxidative stress, cell death, antioxidants, therapeutics, neuroprotection

## Abstract

Traumatic brain injury (TBI) is a major global health problem that affects both civilian and military populations worldwide. Post-injury acute, sub-acute, and chronic progression of secondary injury processes may contribute further to other neurodegenerative diseases. However, there are no approved therapeutic options available that can attenuate TBI-related progressive pathophysiology. Recent advances in preclinical research have identified that mitochondria-centric redox imbalance, bioenergetics failure and calcium dysregulation play a crucial role in secondary injury progression after TBI. Mitochondrial antioxidants play an important role in regulating redox homeostasis. Based on the proven efficacy of preclinical and clinical compounds and targeting numerous pathways to trigger innate antioxidant defense, we may be able to alleviate TBI pathology progression by primarily focusing on preserving post-injury mitochondrial and cerebral function. In this review, we will discuss novel mitochondria-targeted antioxidant compounds, which offer a high capability of successful clinical translation for TBI management in the near future.

## 1. Introduction

Traumatic brain injury (TBI) is caused by a mechanical blow, penetration, bump, or jolt to the head subsequently leading to tissue and cellular damage, and ultimately resulting in alteration of physiological and behavioral functions. TBI represents a major contributor to morbidity and mortality amongst civilian and military populations across the world. There were over 69,000 TBI-related deaths in the United States alone in 2021, accounting for about 190 deaths per day [1]. TBI also has a big global impact, with annual TBI incidence estimated to be 27 to 69 million [2,3]. These injuries have both short-term and long-term effects on individuals, their families, and society and their financial cost is enormous. Many survivors live with significant disabilities, resulting in major socioeconomic burden. The economic impact of TBI in the United States is estimated to be about USD 76.5 billion for survivors [4,5]. Clinically, TBI is categorized as mild, moderate, or severe injury based on the Glasgow Coma Scale (GCS) scores range between 3 to 15, with a lower score indicating more severe brain damage and a poorer prognosis. The GCS describes the level of consciousness of an individual after acute brain trauma [6,7]. Nevertheless, across all TBI severities, the consequences of TBI may lead to long-term disability, including cognitive and motor function limitations/impairments, and decreased psychosocial health.

TBI-induced neuronal tissue damage manifests in primary and secondary injuries. The primary injury stems from the initial mechanical impacts to the brain [8]. The primary injury is considered an irreversible injury resulting from brain tissue compression, displacement, stretching, shearing, tearing, crushing of the brain parenchyma, brain hemorrhage, and blood–brain barrier (BBB) disruption [8]. Following the primary mechanical insult, the downstream sequelae of molecular events activate complex secondary injury pathophysiological cascades such as excitotoxicity, intracranial hypertension, edema, elevated calcium, metabolic dysregulation, mitochondrial dysfunction (energy crisis, antioxidant depletion, and free-radical generation), inflammation, and ischemic injury, which occur at the acute (i.e., minutes to hours) and sub-acute (i.e., hours to weeks) phases of progressive TBI [9,10,11,12]. Consequently, brain functions are first disrupted at the injury site and subsequently disrupted at distal interconnected regions. Despite the advancements in TBI research, the precise mechanisms leading to the progression of TBI pathophysiology are yet to be fully elucidated.

**Figure 1:**
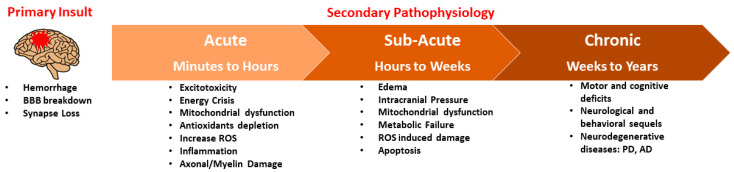
Progressive pathology of TBI. The primary mechanical impact may lead to brain damage in the form of brain hemorrhage, blood–brain barrier (BBB) breakdown and synapse loss, thereby subsequent secondary injury processes beginning immediately after post-injury, and sometimes may sustained over lifetime. During these acute (minutes to hours) and sub-acute (hours to weeks) processes, mitochondria-centric mechanisms play a key role in the further progression of TBI pathology. During the chronic (weeks to years) period of secondary brain injury, neurological behavior deficits in terms of cognitive and motor functions are evident, and may further contribute to neurological diseases such as AD, PD, HD, ALS and MS.

The chronic progression of post-TBI secondary injury responses (i.e., weeks to years) further affects TBI patients’ neuronal ability to maintain their long-term physiological and behavioral functions. TBI progression is linked to the etiology of many neurodegenerative diseases such as Alzheimer’s disease (AD), Amyotrophic lateral sclerosis (ALS), Huntington’s disease (HD), Multiple sclerosis (MS), and Parkinson’s disease (PD) [13]. However, the specific epidemiological factors and pathophysiological mechanisms that underlie this association between TBI and specific neurodegenerative pathologies remain unclear. Notably, reports indicate a 63–96% increased risk of all-cause dementia following TBI [14]. Additionally, the risk of PD may go up by at least 1.8 times following moderate to severe TBI [14,15]. Meta analysis indicates an increased risk of ALS following TBI [16]. Additionally, patients with TBI have a higher risk of developing MS [17]. Furthermore, a World War II study suggested that early-adulthood TBI increases the likelihood of developing AD later in life by 2.3 to 4.5 times, respectively, for moderate and severe injuries [14,15]. These reports suggest that TBI is a major risk factor for the onset of neurodegenerative disorders in later life (Figure 1).

Much of our understanding of the pathobiology of TBI has arisen from animal models that simulate features of human TBI. Multiple preclinical TBI models, including models of penetrating traumatic brain injury (PTBI), controlled cortical impact (CCI) injury, blast-induced traumatic brain injury (BTBI) and closed head injury (CHI) have ascertained that mitochondrial dysfunction is a common and immediate indicator of cellular damage [18,19,20,21] that may even play a critical role in secondary excitotoxic post-injury events. Several detailed previous reports have highlighted preclinical models and cellular mechanisms of TBI [22,23,24,25,26,27,28,29,30]. Mitochondria-centered cellular mechanisms involve calcium homeostasis, energy homeostasis, and redox homeostasis. Their imbalance subsequently may prompt downstream cellular processes such as cell death pathways and neuronal death and alter behavior outcomes in TBI.

Mitochondria are key organelles in all eukaryotic cells and play a central role in cellular energy homeostasis through the metabolism of carbohydrates, fats, and/or proteins. Brain cells manage higher cellular energy (i.e., adenosine triphosphate, ATP) demands by oxidizing their metabolic substrates through respiration and oxidative phosphorylation. Mitochondrial dysfunction following TBI has been shown to be devastating for neuronal cell survival [31,32,33,34]. Several time-course studies of mitochondrial bioenergetics in preclinical models of TBI have suggested that mitochondrial energy failure is the key pathological event that is initiated immediately (e.g., within 30 min) and remains evident for up to 2 weeks after injury [11,12,33,34,35,36].

Interestingly, under normal physiological conditions, intracellular calcium levels are modulated by mitochondria to maintain cellular homeostasis at a certain threshold; however, rapid increase in cellular calcium following TBI may lead to excitotoxicity. Under physiological conditions during ATP production, mitochondria also maintain calcium homeostasis and regulate mitochondrial permeability transition (MPT) pore formation. Several reports have identified impaired mitochondrial calcium-buffering capacity and early mitochondrial MPT opening following acute and sub-acute phases of TBI [37]. Therefore, mitochondrial MPT is considered as the “biological on/off switch” that determines the fate of cells in response to noxious excitotoxic stimuli [38,39,40,41].

Additionally, mitochondrial dysfunction in response to secondary injury elevates the oxidative stress response. Post-TBI oxidative damage leads to structural functional alteration in cellular and subcellular components. This, coupled with the impairment of mitochondrial bioenergetics, initiates a vicious cycle of free-radical formation and apoptosis. In this review, we evaluate the detailed mechanisms of mitochondrial redox homeostasis and discuss potential antioxidant strategies to mitigate oxidative damage following TBI. The aim of this review is to provide a comprehensive overview of antioxidant therapy for TBI to the scientific research community, categorized into different classes, and systematically discussed in the following sections.

## 2. Mitochondrial Redox Mechanisms in TBI

Mitochondria are vital organelles present in all eukaryotic cells, that consume approximately 98% of body’s total oxygen supply. Efficiently utilizing this oxygen, mitochondria produce energy through oxidative phosphorylation processes linked by respiration via the electron transport chain (ETC) complex proteins. In normal physiological conditions, oxygen slippage estimated from 3 to 5% can occur during the utilization of oxygen in the mitochondrial ETC complexes I and III [42,43,44]. This oxygen slippage results in the generation of the superoxide radical (O_2_^•−^), a highly reactive unstable singlet oxygen, which, in turn, can lead to the production of other reactive oxygen species (ROS). Mitochondria also contain antioxidants to manage elevated levels of free radicals as part of normal repair mechanisms. Highly reactive superoxide (O_2_^•−^) is rapidly converted into less-reactive ROS, hydrogen peroxide (H_2_O_2_) by superoxide dismutase (SOD), which is then further decomposed/neutralized into water by catalase (CAT) or peroxiredoxin (Prx) or thioredoxin (Trx) complex enzyme systems. However, O_2_^•−^ also generates hydroxyl (^•^OH) radicals through the Fenton reaction, which can be further converted to peroxynitrite (ONOO^−^), and subsequently other reactive nitrogen species (RNS) such as nitrogen dioxide and peroxynitrous acid (ONOOH). Normally, mitochondria maintain redox homeostasis with antioxidant activities to scavenge ROS–RNS [45,46]. However, under pathophysiological conditions, elevated levels of ROS–RNS have been observed as early as 30 min after TBI [47,48].

Moreover, these harmful ROS–RNS molecules can further oxidize and damage cellular proteins, lipids, nucleic acids, and extracellular matrix components. The oxidized protein adducts, 3-nitrotyrosine (3-NT), protein carbonylation (PC), and the lipid peroxidation adduct 4-hydroxynonenal (4-HNE) are the hallmarks of peroxynitrite-mediated oxidative stress. Additionally, ROS–RNS may further induce nuclear and mitochondrial DNA damage and affect gene expression responses. ROS–RNS overproduction may inducedamage to ETC subunits, which may further exaggerate the vicious cycle of mitochondrial dysfunction.

**Figure 2:**
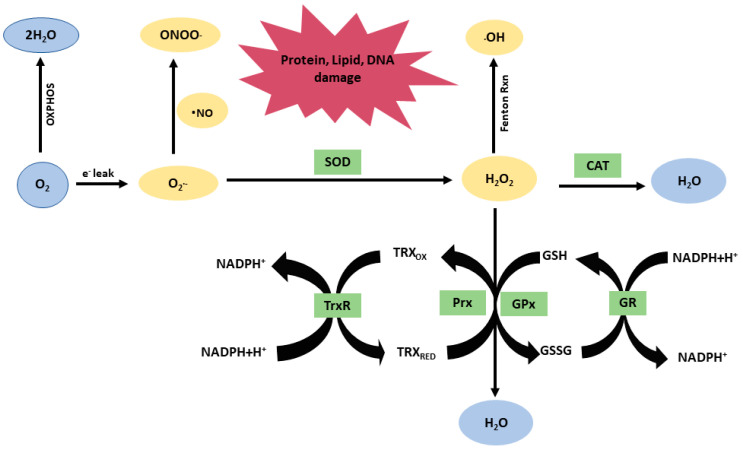
Generation and scavenging of reactive oxygen species (ROS) using the antioxidant defense system. Electrons released from the mitochondrial ETC and produced by NADPH oxidases are the major source of endogenous reactive oxygen species. The oxidative stress generated via this mechanism can be countered via the antioxidant defense system. Oxygen (O_2_) is reduced to superoxide (O_2_•^−^), which can be reduced to hydrogen peroxide (H_2_O_2_) by superoxide dismutase (SOD). Nitric oxide radicals (•NO) form the potent oxidant peroxynitrite (ONOO^−^) following reaction with O_2_•^−^. The H_2_O_2_ can undergo the Fenton reaction and transformed into hydroxyl radicals (•OH) or reduced to water (H_2_O) by catalase (CAT), or the glutathione (GSH)/glutathione peroxidase (GPx) or peroxiredoxin (Prx) systems. The oxidized form of thioredoxin (Trx) is reduced back by the reaction with Trx reductase (TrxR), while that of Grx is reduced back by GSH and terminal NADPH oxidation. An oxidized GSH (GSSG) is reduced back to two GSH molecules through the enzymatic reaction of GSH reductase (GR). Both Trx and Grx reduce protein disulfides. These enzymatic antioxidant defense systems counter free-radical-induced stress, and maintain cellular redox homeostasis. The reactive oxygen species may further lead to protein, lipid, and DNA damage.

Following TBI, higher redox footprints with respect to elevated free radicals (ROS–RNS), together with altered lipid, protein, and DNA adducts have been observed during the acute and sub-acute phases of secondary injury. If not mitigated, these elevated redox mediators may further contribute to other chronic neurological disease pathologies. The protective antioxidant defense system has potential to mitigate the TBI-induced oxidative stress response is further discussed in detail (Figure 2).

## 3. Mitochondrial Antioxidants in TBI

TBI is a highly heterogeneous condition, with patients exhibiting diverse patterns of injury, severity, and outcomes. Oxidative stress plays a crucial role in the development of acute brain injury and acts as a key mediator in the secondary injury cascade of TBI pathology. Oxidative stress leading to oxidative damage represents a state where oxygen levels, combined with oxygen-derived free radicals overwhelm the scavenging antioxidant system.

Following TBI, excitotoxicity occurs, where an excess of Ca^2+^ further promotes ROS–RNS production. The increased ROS–RNS levels, coupled with depleted antioxidant levels after TBI, lead to elevated oxidative stress, wherein protective mechanisms, such as antioxidants, fail to control these radicals, resulting in oxidative stress and subsequent neuronal death. Post TBI, various complications such as brain edema, mitochondrial dysfunction, BBB breakdown, sensory–motor dysfunction, and secondary neuronal injury have been proposed to be linked to oxidative stress [49,50,51]. Our recent findings indicate decreased mitochondrial antioxidants and increased oxidative stress markers during the acute phase of TBI [37], a trend supported by numerous researchers highlighting the role of oxidative stress following TBI [33,52,53,54,55,56,57]. This underscores oxidative stress redox mechanisms as a valid therapeutic target for TBI. Furthermore, antioxidant intervention emerges as a logical therapeutic approach for achieving neuroprotection after TBI. Both elevated free-radical-mediated oxidative stress and depleted endogenous antioxidant responses have been observed during acute and sub-acute phases preclinically; and only limited reports have noted duringchronic phase of TBI [37,52,58].

Antioxidants are substances which scavenge or neutralize free radicals in cells, thereby prevent oxidative damage. Antioxidants may be able to reduce the risk of the onset of chronic diseases. Antioxidant therapy emerge as a novel approach to preventing and treating neurodegenerative conditions where oxidative stress acts as a major contributing factor to the pathogenesis and/or progression of the diseases. There are two main approaches by which antioxidant levels can be replenished in brain cells, and these may serve as options for therapeutic interventions to limit free-radical generation and oxidative stress responses. This, in turn, improves the balance of redox homeostasis after brain trauma. In an injured brain, antioxidants may be able to modulate redox mechanisms through (a) scavenging or detoxifying excessive ROS–RNS using natural or synthetic antioxidants and restrict free-radical overproduction, and (b) modulating cell signaling pathways that favor endogenous antioxidant synthesis and balanced redox homeostasis.

**Figure 3:**
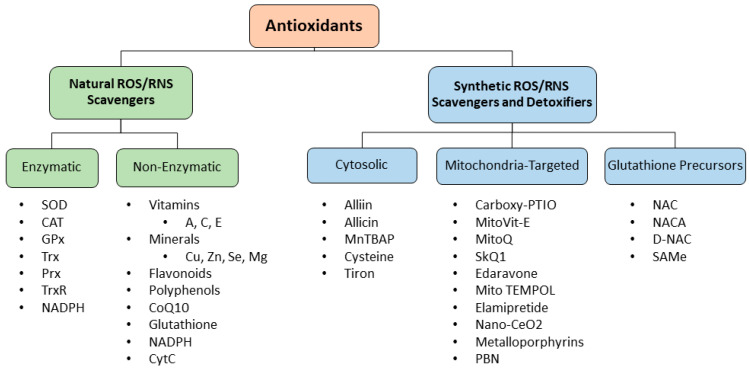
Classification of different antioxidants discussed in the current review. As shown in this illustration, antioxidants are further classified into natural and synthetic ROS–RNS scavengers and detoxifiers. Among the natural antioxidants, they are further sub-divided into enzymatic and non-enzymatic forms, whereas in the group of synthetic antioxidants, they are further sub-divided into three categories, namely non-targeted cytosolic, mitochondria-targeted, and glutathione precursors, based on their sub-cellular target, pharmacological properties, and abundance. Examples of each category of antioxidants are listed and discussed in detail.

This review highlights each category of antioxidants that may serve as future therapeutic options to restrict/stimulate the mechanisms listed above and favor balanced redox homeostasis following the secondary injury phases of TBI. Unfortunately, there are no FDA-approved treatment options that are currently available to restrain multifaceted TBI pathophysiology, leaving a critical gap unfilled. Therefore, more preclinical research efforts are warranted to identify novel therapeutic targets. Additionally, repetitive injuries aggravate secondary injuries and lead to early neurological deficit. Collaborative efforts between preclinical and clinical communities under regulatory guidance of the FDA are ongoing to conduct better-designed clinical studies, and gain rapid approvals of therapeutic products for TBI. This review is intended to provide an overview on comprehensive information about antioxidant therapy for TBI to the scientific research community, classified into different categories (Figure 3), and discussed below.

## 4. ROS–RNS Scavengers

The ROS–RNS scavengers/detoxifiers are further categorized as natural (e.g., endogenous enzymatic or non-enzymatic) and synthetic (e.g., drug molecules and dietary supplements), as described below.

### 4.1. Natural ROS–RNS Scavengers

Among natural enzymatic antioxidants, the mitochondria-specific superoxide dismutase (SOD) isoform (e.g., manganese SOD or Mn-SOD) plays a critical role in scavenging mitochondrial production of O_2_^•−^ at the mitochondrial ETC complex I and III sites [59,60]. Another isoform of SOD (i.e., cytosolic copper–zinc SOD or Cu-Zn-SOD) scavenges O_2_^•−^ in the cytosolic compartment. The mitochondrial ETC is a primary site of O_2_^•−^ generation; therefore, scavenging O_2_^•−^ at the mitochondrial ETC level offers the greatest benefit. Catalase (CAT) is an additional important ROS scavenger that neutralizes H_2_O_2_ and converts it into water [61]. The redoxin family, including peroxiredoxin (Prx) and thioredoxin (Trx) enzyme systems, together with other non-enzymatic reducing cofactors, nicotinamide adenine dinucleotide phosphate (NADPH) and/or glutathione, also plays an important role in scavenging ROS [37,61]. They help to convert H_2_O_2_ into water (Table 1, and Figure 2).

Chronic exposure to oxidative stress may further lead to activation of the first line of antioxidant defense by increasing SOD-, CAT-, and GPx-mediated protective feedback mechanisms that may be able to help mitigate oxidative stress responses to some extent. Interestingly, we observed a depletion of SOD protein expression during the acute period following PTBI [37]. Other studies have also detected diminished SOD activity during the acute phase of TBI that remained low for at least several weeks post TBI [60,62]. Depletion of SOD after TBI could make injured tissue more susceptible to increased O_2_^•−^ formation, amplifying post-injury oxidative damage over time. In contrast, studies have reported an increase in CAT during the acute and sub-acute phases following TBI; however, the precise mechanism by which brain injury leads to increased CAT protein expression is currently unknown [37]. Earlier studies have reported decreased SOD, CAT, and GPx antioxidant enzyme activity in AD patients [63,64]. Interestingly, in an AD mouse model, the overexpression of the SOD protein showed great promise in relation to improvement in neurological outcomes [65]. Therefore, in therapeutic applications, SOD and SOD mimetics have great potential to serve as a drug to ameliorate TBI-related oxidative damage.

The other class of natural antioxidants are the non-enzymatic antioxidants, which are mainly acquired from dietary sources; these are also called natural ROS scavengers. The most common dietarily derived antioxidants are Vitamin A (retinol), Vitamin C (ascorbate), Vitamin E (α-tocopherol), carotenoids (carotene, zeaxanthin, lutein, lycopene, cryptoxanthin, retinoids), polyphenols, and flavonoids, among others.

Vitamin A is obtained from dietary sources such as green and yellow vegetables, dairy products, fruits, and meats. Vitamin A can act as a chain-breaking antioxidant by combining with reactive radicals before these radicals can propagate peroxidation in the lipid phase of the cell and generate H_2_O_2_ [66]. Likewise, Vitamin C is acquired from dietary sources such as fruits and vegetables, and is available as a dietary supplement. Vitamin C has been used as an antioxidant to treat mitochondrial diseases; additionally, it can act as an electron transfer mediator to bypass complex III in combination with Vitamin K at the ETC [67,68]. The oxidized form of Vitamin C is transported into the mitochondria via glucose transporter 1, which helps to maintain a healthy mitochondrial membrane potential and inhibits mitochondrial membrane depolarization [69]. Additionally, Vitamin C facilitates electron movement, favoring energy production [70]. Vitamin E is mainly found in vegetable oil and its derivatives, nuts and seeds. Vitamin E interrupts the chain reaction of oxidant generation and oxidative damage by capturing free radicals.

Carotenoids are another class of antioxidants, and their main dietary sources are red vegetables and fruits (carrots, tomatoes, apricots, plums) and green leafy vegetables (spinach, kale). Indeed, carotenoids are important precursors of Vitamin A [71]. Carotenoids are very efficient quenchers of singlet oxygen and potent scavengers of other ROS–RNS. Similarly, polyphenol antioxidants (flavanols, anthocyanins, isoflavones, phenolic acid), mainly found in fruits (apples, berries, grapes), vegetables (celery, kale, onions), legumes (beans, soybeans), nuts, wine, tea, coffee, and cocoa, can be obtained from nutritional sources. Polyphenol acts as an antioxidant via a direct ROS-scavenging mechanism and the modulation of antioxidant enzymes. Flavonoids are phenolic structures containing natural substances mainly found in fruits, vegetables, grains, bark, roots, stems, flowers, tea, and wine. Flavonoids exert antioxidant, anti-inflammatory, and anti-cholinesterase activities. Flavonoids act as potent inhibitors for several enzymes, such as xanthine oxidase (XO), cyclo-oxygenase (COX), lipoxygenase, and phosphoinositide 3-kinase [72,73]. Pycnogenol (PYC) is a combination of bioflavonoids that is extracted from the bark of the French maritime pine tree (Pinus maritima), and has a robust capacity to scavenge free radicals. The neuroprotective effects of PYC have been explored in a rodent model of TBI [74]. Additionally, alliin, a garlic-derivative compound, reacts with O_2_^•−^ and scavenges by utilizing the xanthine/xanthine oxidase system [75]. Allicin, a derivative of alliin, also inhibits O_2_^•−^, nitric oxide (NO^•^), and hydroxyl (^•^OH) radical production [75,76,77].

Some antioxidants are produced by cells that chelate and/or bind to redox metals, thus protecting the cells against oxidative stress indirectly. Micronutrients such as metal and trace elements (zinc, iron, selenium, and copper) possess antioxidant properties. Supplementation with either selenium or zinc has been found to restore the alterations of mitochondrial parameters, including ETC enzymes and antioxidant enzymes, in several diseases [78].

The membrane-bound coenzyme Q10 (CoQ10) is an important antioxidant that is part of the mitochondrial ETC. It shuttles electrons from complexes I/II to complex III. CoQ10 prevents the generation of free radicals and modifications of proteins, lipids, and DNA. Thus, CoQ10 markedly regulates the cellular redox balance.

Among other non-enzymatic ROS scavengers, one of the key cellular antioxidants is glutathione (e.g., γ-L-glutamyl-L-cysteinylglycine). Glutathione is synthesized from the amino acids L-cysteine, L-glutamic acid, and glycine. Glutathione is an important antioxidant which reacts with ROS using thiol-SH groups of cysteine. Glutathione is a ubiquitously distributed tripeptide antioxidant abundantly present in all cells in millimolar concentrations (~5 mM) [79]. The reduced form of glutathione (i.e., GSH) is involved in various cell functions, including the detoxification of oxidized amino acids/proteins, the biosynthesis of proteins and DNA precursors, amino acid transport, and the maintenance of redox balance. During this process, the endogenously generated oxidized glutathione (GSSG) can be recycled back to GSH by the endogenous Grx system. The GSH/GSSG ratio remains an important indicator of redox homeostasis and imbalance in cell oxidative metabolism.

Another antioxidant, NADPH (e.g., nicotinamide dinucleotide phosphate), works closely with glutathione and other redoxin enzymes to protect against ROS–RNS-induced cell damage. In redoxin systems, NADPH serves as a cofactor, used for transferring and preserving redox potential for multiple antioxidants such as glutathione, Prx, and Trx. This NADPH-induced conversion reactivates the functions of antioxidant molecules. We found that NADPH levels significantly decreased following TBI [37]. This reinforces the importance of exogenous NADPH treatment following TBI to increase the effectiveness of antioxidant proteins as the scavengers of oxidants. Additionally, in cells, endogenous cytochrome C (Cyt C) may act as an O_2_^•−^ scavenger since it is reduced by O_2_^•−^ and oxidized by H_2_O_2_ [80]. Cyt C seems to be an ideal antioxidant since Cyt C can regenerate and avoid being damaged during antioxidant reactions [81].

Additionally, several dietary or nutritional supplements serve as conventional (non-targeted) antioxidants in cells. However, all of these natural antioxidants have limited effectiveness in scavenging mitochondrial ROS–RNS and oxidative stress due to their limited ability to cross the mitochondrial biomembranes [82,83]. In the next section, we compile a list of mitochondria-targeted synthetic antioxidants, which may serve as better options to combat ROS–RNS and oxidative stress and may offer neuroprotection.

### 4.2. Synthetic ROS–RNS Scavengers

Novel synthetic ROS–RNS scavengers targeted towards preventing or minimizing oxidative damage have contributed new insights into potential neuroprotective therapies (Table 2). Superoxide (O_2_^•−^) scavengers are important antioxidants due to their ability to mitigate oxidative stress during the acute post-injury phase. One such synthetic compound is Mn (III) tetrakis (4-benzoic acid) porphyrin (MnTBAP), an ROS scavenger. MnTBAP is both an SOD mimetic and peroxynitrite (ONOO^−^) scavenger [84,85]. Other compounds that can donate electrons to O_2_^•−^ are ascorbic acid, cysteine (via the sulfhydryl group), tiron, and carboxy-PTIO (a nitric oxide scavenger), which can also react with superoxide radicals [86,87,88,89,90,91,92,93,94]. Tiron is a Vitamin E-analog antioxidant that can enhance NF-κB-dependent gene transcription with an anti-apoptotic effect [95]. Carboxy-PTIO is an imidazole-derived free-radical scavenging compound that inactivates NO^•^ and NO_2_, subsequently reacting with water to form nitrite and nitrate. Phenelzine (PZ) is an FDA-approved drug for the management of treatment-resistant depression, panic disorder, and social anxiety disorder that functions as an MAO inhibitor [96]. PZ has aldehyde-scavenging properties. PZ administration was also shown to significantly improve mitochondrial respiration following TBI [96].

Moreover, to overcome the limited effectiveness of natural ROS scavengers, several synthetic mitochondrial ROS scavengers have been designed to cross the BBB and accumulate in neuronal mitochondria. These compounds are formulated to target mitochondria at the injured region to neutralize ROS and promote the mitigation of oxidative damage, together with improving bioenergetic function. The development of antioxidants capable of restoring mitochondrial function following brain injury is highly significant since redox homeostasis dysregulation is a critical factor in the cell death pathway during the acute, sub-acute, and chronic phases of TBI. Also, specific mitochondrial targeting leads to more precise and effective mitigation of redox homeostasis. We have compiled a list of such covalently modified compounds in this table.

The synthetic mitochondrial-targeted Vitamin E compound (MitoVit-E) is created by covalently attaching natural Vitamin E (α-tocopherol) to a triphenylphosphonium (TPP^+^) cation. MitoVit-E facilitates the accumulation of TPP^+^ in the mitochondrial matrix against the negatively charged mitochondrial membrane potential (ΔΨm). This unique feature makes MitoVit-E an effective mitochondria-targeted ROS scavenger. By utilizing the concentration gradients of ΔΨm, MitoVit-E decreases ROS production and apoptosis in aortic endothelial cells via peroxide-induced oxidative stress and apoptosis [97,98]. One disadvantage of Vitamin E is that it is not a catalytic antioxidant and therefore its scavenging activity is not regenerated. Another well-studied mitochondria-targeted antioxidant is mitoquinone (MitoQ), a ubiquinone derivative conjugated to the TPP^+^ cation that serves as a potent reactive oxygen species (ROS) scavenger. MitoQ has structural similarity with endogenous components of the mitochondrial ETC ubiquinone; therefore, it may help in assisting efficient electron transfer through the ETC [99,100]. The active form of MitoQ, i.e., ubiquinolis able to scavenge ROS and is being modified into its inactive form, ubiquinone. This inactive form is continuously recycled back into its active form by the mitochondrial complex II. This reduction–oxidation cycle enables MitoQ to maintain an efficient chain-breaking antioxidant capacity. MitoQ treatment has been shown to inhibit mitochondrial oxidative damage in rodent models of cardiac ischemia and reperfusion injury [101]. The antioxidant properties of MitoQ were further demonstrated in several preclinical models of TBI, where it increased the activity of antioxidant enzymes and reduced oxidative damage [102,103]. Plastoquinonyl-decyl-triphenylphosphonium bromide (SkQ1) is another class of mitochondria-targeted antioxidant [104]. In the case of SkQ1, the phosphorus cation bound to three phenyl rings (TPP^+^) is conjugated to plastoquinol via a decyl linker. The binding of this cation to the phenyls ensures the ability of SkQ1 to penetrate membranes [105]. A positive electrical charge leads to a thousand-fold accumulation of SkQ1 in the mitochondrial membrane’s inner layer [105]. SkQ1 is able to reduce cardiac ischemic injury, and is well known for lipid peroxidation inhibition [106,107,108,109].

Edaravone is a free-radical scavenger that can quench hydroxyl radicals and hydroxyl radical-dependent lipid peroxidation. It is an FDA-approved compound for the treatment of acute ischemic strokes and amyotrophic lateral sclerosis (ALS) [110]. Additionally, edaravone has shown promising beneficial effects in a wide range of diseases, such as PD, AD, atherosclerosis, chronic heart failure, and diabetes mellitus [111,112,113,114]. Other potential synthetic antioxidants designed to reduce oxidative damage effectively include Mito TEMPOL, elamipretide (SS-31), cerium oxide nanoparticles (Nano-CeO_2_), metalloporphyrins, and phenyl-tert-butylnitrone (PBN) [115,116,117,118,119,120,121]. However, their roles in TBI have not yet been investigated. Research on the evaluation of ROS–RNS scavengers in TBI is ongoing, and the field continues to explore novel approaches and compounds to mitigate oxidative stress and improve behavioral outcomes following TBI [52,58,122,123]. Indeed, several preclinical studies have shown the therapeutic efficacy of mitochondria-targeted antioxidants by improving cognitive and functional recovery post TBI [122,123]. Thus, this strategy may offer new hope for treating TBI patients.

Amongst synthetic ROS scavengers and detoxifiers, novel precursors of glutathione play a significant role. Glutathione, a ubiquitous reducing sulfhydryl tripeptide, plays a major role in ROS–RNS detoxification. Many studies have reported a depletion of glutathione and its precursors, namely cysteine, methionine, and glycine, in brain tissue and cerebrospinal fluid (CSF) following TBI [37,49,50,124]. Therefore, several strategies have explored boosting glutathione levels following TBI to protect neurons against oxidative damage. One approach is to administer glutathione directly. Glutathione injections have been used in the past to boost glutathione levels in blood and skin; however, there was no systemic study available to prove its efficacy [125]. Direct enhancement of glutathione comes with its own challenges like short half-life, absorption, BBB permeability, and limited brain bioavailability [125].

The de novo synthesis of glutathione is primarily controlled by the cellular concentration of cysteine. In keeping with this, NAC and its analogs, such as the cysteine supplement, are effective at raising levels of glutathione in various neurological diseases and injuries, preclinically and clinically [126,127,128,129]. Therefore, various glutathione prodrugs or antioxidant supplements to boost innate glutathione levels have been investigated.

N-acetyl cysteine (NAC) is perhaps the most widely studied glutathione precursor to act as an antioxidant. NAC has been approved by the FDA for treating hepatotoxic doses of acetaminophen (Tylenol). Additionally, NAC has been widely used because of its mucolytic effects, taking part in the therapeutic protocols of cystic fibrosis. Over the past decade, studies have documented the positive outcome of NAC treatment for many CNS diseases, including TBI [130]. Additionally, NAC’s ability to replenish glutathione, maintain cellular homeostasis, and support mitochondrial function has been successfully demonstrated in TBI [131,132,133,134,135].

Clinical treatment with NAC has been shown to upregulate glutathione-centered pathways in the CSF of severe TBI pediatric patients (ClinicalTrials.gov NCT01322009) [136,137,138]. NAC treatment was evaluated in U.S. service members who had been exposed to a blast-induced mild TBI [139]: the outcome of this study demonstrated NAC as safe and effective pharmaceutical agent for acute countermeasure. NAC treatment has beneficial effects on the injury severity, and resolution of post-traumatic sequelae of blast-induced mild TBI (ClinicalTrials.gov NCT00822263) [139]. Furthermore, NAC’s neuroprotective effects are mediated by both antioxidant and anti-inflammatory mechanisms [140,141,142,143]. These multimodal neuroprotective properties of NAC may confer significant benefits on the complex and heterogenous nature of TBI pathology.

The BBB permeability of NAC is limited by its physiochemical properties, such as its acidic nature and negative charge [144,145]. Notably, numerous studies evaluating the neuroprotective properties of NAC have yielded inconsistent results, which may be due to its low bioavailability [144,145]. A potential strategy for overcoming the low bioavailability of NAC is to use an NAC analog where the carboxyl group of NAC is neutralized, thus making it more hydrophobic and increasing its BBB permeability [146]. In this regard, the preparation of NAC analogs, such as N-acetylcysteine amide (NACA), is very attractive and may have advantages over NAC in treating CNS pathologies due to the improved stability and bioavailability [147]. For instance, there are studies reporting the neuroprotective efficacy of NACA in neurological diseases including PD, AD, and HIV-associated neurological disorders [148,149,150]. In the same line of effort, we have demonstrated that NACA effectively reduces oxidative damage, maintains the glutathione level, and improves mitochondrial bioenergetics following TBI [128,129,151]. Other studies have reported similar outcomes in spinal cord injury (SCI) patients [129]. Thus, NACA may offer neuronal protection by reducing oxidative stress and supporting cellular pathways to limit mitochondrial dysfunctions following TBI.

To enhance NAC’s bioavailability and address neurological conditions, researchers are investigating an alternative intranasal route for its direct delivery to the CNS via neuronal pathways, thus minimizing the BBB permeability issues [152]. However, the optimal dosing regimen for this intranasal route of NAC administration still needs to be further investigated at the preclinical level for TBI.

Recently, researchers have used nanoparticle delivery systems, such as dendrimers, to ensure targeted and effective drug delivery to the CNS. Hydroxyl-terminated polyamidoamine (PAMAM) dendrimer, a dendrimer linked with NAC (D-NAC), has shown to be a promising route of drug delivery to injury sites within the brain. In particular, D-NAC has been investigated as a drug delivery system to target cells involved in neuroinflammation [153]. In the presence of a brain injury, D-NAC traverses the BBB and localizes specifically in activated microglia and astrocytes, and the extent of its uptake correlates with the extent of the injury [154,155]. D-NAC also has been shown to be effective in improving myelination and motor functions in cerebral palsy [156,157]. The protective role of D-NAC has been established in ischemic brain injury, asphyxia brain injury [158,159,160], and other CNS pathologies like choroidal and retinal neovascularization [161]. Collectively, novel dendrimer-based delivery methods, such as D-NAC, appear to be promising avenues for targeting therapeutic agents in CNS diseases.

Similarly, another compound that aids in restoring glutathione synthesis by recycling its precursor cysteine is S-adenosyl methionine (SAMe). SAMe has been studied for its potential neuroprotective efficacy in several CNS diseases [162,163]. Besides providing amino acids during methyltransferase reactions for glutathione synthesis, SAMe serves as a key metabolite in many biochemical reactions, and is available as a dietary supplement. Depletion of methionine and its crucial metabolites has been reported in TBI; therefore, restoring methionine metabolites with SAMe supplementation may improve its outcome [124].

A thorough understanding of methods to replenish glutathione and the application of innovative technology to advance targeted therapy in research is critically important when considering therapies to combat TBI secondary pathogenesis. Similarly, the development of neuroprotective formulations to enhance signaling pathways to upsurge innate antioxidants as a potential tool for the therapeutic treatment of neurological diseases represents an important goal for current neuroscience research.

## 5. Signaling Pathway Modulators for Cellular Antioxidant Synthesis

**Figure 4:**
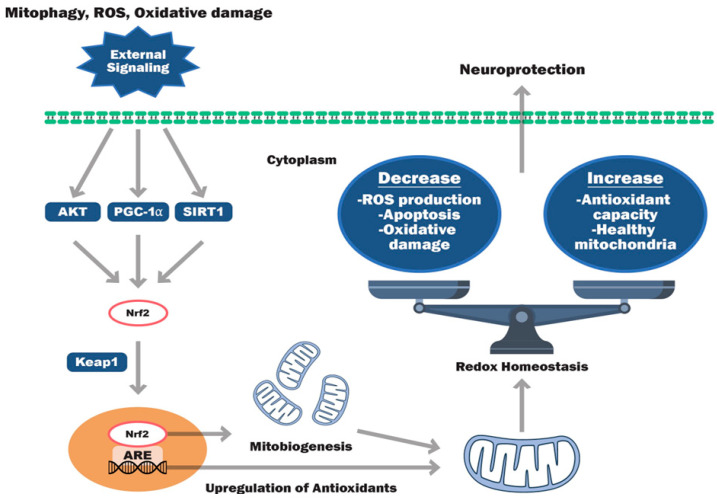
Illustration of signaling pathway modulators involved in endogenous cellular antioxidant synthesis. Extracellular stimulants such as mitophagy, ROS, and oxidative damage may lead to the activation of cell signaling pathways such as AKT, PGC-1α, mTOR, and SIRT. These signaling pathways are involved in upregulating endogenous antioxidant homeostasis by activating nuclear antioxidant response element (ARE) signaling via the activation of common transcription factors Nrf-2 and Keap1. Activation of the nuclear ARE gene upregulates mitochondrial biogenesis. Additionally, ARE gene expression activation may also leads to activation of several mitochondrial antioxidant transcription factors, thereby protein biosynthesis and protect cells against external stimuli. Increased antioxidant levels further balance redox homeostasis by decreasing cellular ROS, oxidative stress, and apoptotic cell death response together by improving the cellular antioxidant capacity and overall health of mitochondria. By activating these pathways, therapeutic compounds may be further able to offer neuroprotection following TBI and CNS diseases.

The initiation of redox homeostasis originates from extracellular or intracellular signals via nuclear receptors and mitochondria-mediated pathways. There are intra- and extracellular signaling pathways that activate the protective mechanisms that particularly trigger the endogenous synthesis of antioxidants. Inducers such as ROS, oxidative stress, mitophagy, apoptosis, excitotoxicity, ischemic insults, calcium, neurotransmitters, exercise, or therapeutic treatment (agonists/antagonists) may trigger the onset of signal transduction via modulating several transcription factors in the nucleus, thereby activating gene expression of downstream protein expression. More specific to the current review topic, there are several inducers listed below that may be able to modulate notable antioxidant signaling pathways, such as the Nrf2, AKT, SIRT1, PGC1α, and mTOR signaling pathways (Table 3). The Nrf2 pathway centers around the broad-reaching transcription factor Nrf2, which modulates the transcription of a myriad of endogenous antioxidants. Protein kinase B, a serine/threonine kinase (AKT), is the main mediator of the downstream effector protein phosphoinositide 3-kinase (PI3K). AKT serves as the central component in numerous signaling pathways regulating cell metabolism, growth, proliferation, and survival. Thus, activating AKT can help preserve typical mitochondrial function across several disease conditions [164]. Additionally, AKT regulates Nrf2 to affect the transcription of pro- and antioxidant enzymes and maintain the cellular redox state [165]. Likewise, SIRT1 is a deacetylase that controls the expression of a multitude of antioxidants and oxidative stress modulators like PGC-1α, which plays a major role in the antioxidant defense system. The rapamycin (mTOR) signaling pathway integrates both intracellular and extracellular signals, and serves as a regulator of cellular metabolism, growth, proliferation, and survival. These pathways, in turn, modulate gene expression and the protein biosynthesis of downstream targets, such as antioxidants and mitochondrial biogenesis proteins. Thus, these pathways may be able to modulate ROS–RNS levels, keep the redox balance in check, and maintain cellular integrity. An overview of cell signaling pathways favoring cellular antioxidants synthesis and neuroprotection is illustrated in detail (Figure 4) and discussed below.

### 5.1. Nrf2 Activators

One of the main cellular signaling and oxidative stress defense pathways is the nuclear factor erythroid 2 (Nrf2)-dependent transcriptional mechanism. Nrf2 is responsible for regulating an extensive panel of antioxidant enzymes involved in the detoxification of oxidative stress. Several strategies have been proposed to activate this pathway to counter ROS production and promote neuroprotection. Nrf2 is a transcription factor responsible for regulating the expression of various downstream genes that modulate the oxidative stress response through regulating the antioxidant response element (ARE). Thus, Nrf2-targeted genes affect many vital antioxidants through ARE gene regulation, such as SOD, CAT, and GPX, among others, which help in combatting ROS [166]. Additionally, Nrf2 downregulation supports the decreased efficiency of mitochondrial oxidative phosphorylation. It is widely thought that the Nrf2 pathway plays an important role in TBI pathogenesis, and even in other neurological diseases like AD and PD [167,168,169]. In a mouse model of TBI, Nrf2 was found to be downregulated in cortical tissue, leading to increased oxidative stress, inflammation, and apoptosis [168]. Therefore, targeting and activating Nrf2 signaling is a potential novel target following the oxidative stress-centered pathology of TBI. Owing to its excellent therapeutic potential in CNS diseases in both preclinical and clinical settings, recently, several Nrf2 activators have been approved by the FDA.

Omaveloxolone (RTA-408) and dimethyl fumarate (DMF) are both FDA-approved Nrf2 activators used to treat various neurological conditions like FA and MS. Other Nrf2 activators like curcumin, sulforaphane, epigallocatechin gallate (EGCG), quercetin, oltipraz, and bardoxolone methyl also hold promise as therapeutic agents due to their antioxidant properties [170,171].

RTA-408 is one of the FDA-approved Nrf2 activators for treating FA, a progressive neurodegenerative condition. RTA-408 affects the Nrf2 pathway by preventing Nrf2 ubiquitination and degradation, leading to Nrf2 translocation to the nucleus and increased antioxidant expression. RTA-408 therapy has been found to enhance mitochondrial function and improve neurological symptoms, cognitive impairment, and neuroinflammation in multiple preclinical and clinical models of CNS conditions like epilepsy [172]. RTA-408’s efficacy in minimizing CNS pathology and its mitochondria-protective properties makes it a potential candidate for treating TBI and other neurological diseases [173,174,175].

Similarly, dimethyl fumarate (DMF) is another Nrf2 activator approved by the FDA for treating MS [176,177]. DMF influences the Nrf2 pathway by modifying Keap1, thus promoting Nrf2 nuclear translocation. DMF also activates AKT pathways and thus promotes neuroprotection [178]. DMF upregulates several antioxidants including glutathione, bolstering the downstream antioxidant capacity in CNS conditions like MS, cerebral edema, TBI, and intracerebral hemorrhage [179,180,181,182,183]. DMF treatment in clinical settings has shown long-term efficacy in reducing relapse rates and minimizing lesion formation in relapsing forms of MS [184]. Furthermore, DMF treatment was found to increase neuronal mitochondrial biogenesis via Nrf2 regulation along with improved mitochondrial function and neurological symptoms in a preclinical model of MS [185]. Additionally, DMF has shown to improve cognitive functions in animal models of AD and PD [186]. Together, this evidence emphasizes that DMF has broader therapeutic applicationfor MS, and other neurodegenerative diseases.

Several Nrf2 activators listed here exhibit potential health benefits. Curcumin, found in turmeric, a commonly used spice in Indian cuisine and in traditional medicine, activates the Nrf2 pathway, leading to increased antioxidant and detoxifying enzyme production [187]. Curcumin has shown to be effective against cancer, cardiovascular diseases, and various metabolic and neurological conditions [188]. Sulforaphane, another compound, is mainly found in cruciferous vegetables such as broccoli, cabbage, and brussels sprouts. It activates Nrf2 by inhibiting the protein Keap1 [189]. It similarly enhances endogenous antioxidants and detoxifying enzymes. Sulforaphane has shown therapeutic potential against neurodegenerative diseases [190]. Epigallocatechin gallate (EGCG) is a catechin present in green tea. It activates Nrf2, and it is known for its antioxidant and health-promoting properties [191]. Research suggests that ECCG may help protect neurons from oxidative damage and improve cognitive function [192]. Quercetin, found in fruits and vegetables, is an Nrf2 pathway activator that reduces inflammation and improves antioxidant defense mechanisms. The therapeutic effects of quercetin have been investigated in cancer, cardiovascular diseases, and neurodegenerative conditions [193,194]. Oltipraz, a synthetic compound, activates Nrf2 and reduces oxidative stress in cancer, and additionally has shown neuroprotective benefits [195]. Bardoxolone methyl, another synthetic compound, activates the Nrf2 pathway and stimulates antioxidant enzyme production [196,197]. It has been found to be effective against various disease conditions. Although Nrf2 compounds have shown promising protective effects in various health conditions, further research is warranted to confirm and fully understand their safety and efficacy.

### 5.2. SIRT, PGC-1α, AKT, and mTOR Modulators

There are other critical regulatory mechanisms in redox homeostasis, such as the Silent Information Regulator (SIRT) genes, also known as Sirtuins, which stimulate antioxidant expression of several enzymes. One of the members of the SIRT family, SIRT1, is a nicotinamide adenine dinucleotide (NAD^+^)-dependent deacetylase that plays a wide range of roles in transcriptional regulation, inflammation, cell survival, and repair mechanisms. It guards against oxidative stress by activating the gene transcription of peroxisome proliferator-activated receptor gamma coactivator-1α (PGC-1α) via the removal of the acetyl group [198]. PGC-1α is a transcriptional coactivator that is able to upregulate mitochondrial biogenesis, and plays a central role in regulating the oxidative stress defense [199]. SIRT1 is described as a complex target for multiple strategies addressed for the prevention/treatment of several chronic age-related diseases and CNS diseases. Natural and synthetic SIRT1 modulators have been examined. This review examines compounds of a natural origin that have recently been found to upregulate SIRT1 activity, such as polyphenolic products in fruits, vegetables, and plants, including resveratrol, quercetin, and curcumin.

Resveratrol is a natural polyphenol found in various plant sources, such as grapes, berries, and peanuts, which acts as an antioxidant by activating SIRT1. SIRT1 is involved in various cellular processes, including mitochondrial biogenesis [200,201,202]. Resveratrol also activates the Nrf2 signaling pathway to ameliorate oxidative stress and improve mitochondrial function [203]. Moreover, resveratrol activates the PI3K/AKT pathway. On the other hand, resveratrol modulates the recently identified mammalian target of the rapamycin (mTOR) and Janus kinase/signal transducer and activator of transcription (Jak/STAT) pathways to enhance antioxidant defense and positively modulate mitochondrial function. Resveratrol has been suggested to influence mitochondrial dynamics by modulating the balance between mitochondrial fusion and fission, thus regulating mitophagy. Proper regulation of fusion/fission processes is crucial for maintaining mitochondrial health and function. Therefore, resveratrol helps preserve mitochondrial integrity.

Moreover, resveratrol has been shown to be beneficial in neurological diseases like AD, PD, HD, and ALS [201,202,204]. The evidence supports resveratrol’s role in attenuating TBI-associated behavioral abnormalities, brain edema, and pathophysiology [205,206,207,208,209,210]. In a TBI preclinical model, resveratrol improved mitochondrial biogenesis and function by activating the PGC-1α signaling pathway [210]. PGC-1α is central modulator of cell metabolism, where it regulates mitochondrial biogenesis and oxidative metabolism, and controls the expression of antioxidants. It is important to note that while numerous preclinical studies and some clinical trials have explored the potential benefits of resveratrol, the findings are often mixed, and the optimal dose and duration of resveratrol supplementation for specific health conditions remain areas of ongoing research. Other compounds like berberine and metformin which activate PGC-1α may be more useful in neurodegenerative diseases conditions [211,212,213,214].

Despite the potential positive health benefits of resveratrol, it exhibits low CNS bioavailability. This unfavorable pharmacokinetic profile of natural SIRT1 modulators has prompted the development of novel compounds that can positively modulate SIRT1 activity and display better neuroprotective efficacy profiles. Numerous synthetic SIRT1 modulators have been formulated, such as SRT2104, 1,4-dihydropyridine derivative, naphthofuran derivative, and bisarylaniline derivative. However, studies to confirm the pharmacokinetic profiles of these compounds are ongoing. These compounds may have implications in CNS therapeutic development. Additionally, mTOR modulators like everolimus and temsirolimus might regulate ROS through mTOR-mediated antioxidant defense [215]. The mTOR signaling pathway is at the core of many metabolic activities; its activation improves oxidative stress adaptation by activating Nrf2-associated antioxidant signaling [216].

## 6. Challenges and Future Approach

Despite significant strides in characterizing TBI pathophysiology and identifying therapeutic interventions, the landscape is marred by numerous clinical trial failures, even after promising preclinical success [217,218,219,220]. Several conceptual and methodological issues have undoubtedly contributed to the hitches in translating the preclinical results of antioxidant therapy to a clinical setting. Major challenges lie in the inherent heterogeneity of traumatic brain injuries, the complex and multifaced pathology of brain injuries, the limited information on their molecular pathology, the clinical predictiveness/relevance of animal models, the adequacy of pharmacological methodology, the ill-defined category of TBI, and the outcome measures used. Herein, we reviewed some of these critical problems and potential solutions.

The complex and multifaceted nature of TBI pathophysiology complicates treatment, rendering it challenging to address comprehensively with a single targeted drug. A drug targeting multiple components of the secondary TBI cascade may have superior potency compared to a drug that has a single target. For instance, the Nrf2 pathway activator discussed above broadly modulates intracellular and mitochondria-mediated oxidative and inflammatory responses and may support multiple innate defense mechanisms against TBI pathology. Therefore, any drug with pleiotropic mechanisms of action may be advantageous for TBI research [221].

It has been suggested that the complex pathophysiology of TBI may even possibly be addressed through a combination of therapeutic interventions [222]. The need for integrated multitargeted treatments for TBI has been recognized [223]. At the mitochondrial level, we have identified significant impairment of multitargeted homeostasis, including bioenergetics, calcium, apoptosis, and redox mechanisms, post TBI [11,37]. Providing acetate supplements such as glyceryl triacetate (GTA) and acetyl-L-carnitine (ALC) to boost energy production could contribute to neuronal repair and recovery in the energy deprivation-related pathophysiology of TBI [224,225]. Combining acetate therapy with antioxidants may have additive or synergetic mitochondrial mechanism-targeted neuroprotective efficacy compared to monotherapy in attenuating TBI pathology or promoting recovery. Thus, an effective approach to interrupt post-injury oxidative brain damage might involve the combined treatment of antioxidants with mechanistically complementary energy substrates that simultaneously provide a boost in their antioxidant capacity. Ideally, numerous combination therapies should undergo preclinical testing, with the best combinations chosen for further clinical exploration. An efficient and validated screening platform for candidate therapeutics, sensitive and clinically relevant biomarkers and outcome measures, and standardization and data sharing across centers would greatly facilitate the development of successful combination therapies for TBI [221].

There remains a strong need for rigorous studies to understand the temporal profile of oxidative injury mechanisms following preclinical heterogeneous models of TBI, which may identify novel targets for evaluating neuroprotective therapeutics. As the pathophysiology of secondary injury evolves over time, antioxidant interventions must be able to adapt to evolution in the molecular causes of injury; each compound is likely to have a unique therapeutic time window based on the molecular timeline of secondary injury, during which it is most effective and outside of which it may lack significant benefit [222]. Thus, it is crucial to determine the most efficacious therapeutic window for initiating each antioxidant based on its physiochemical properties and molecular targets in TBI.

Many pharmacological methodological issues have limited the clinical application of antioxidant therapies. Failure to demonstrate sufficient CNS penetration, inadequate dose optimization, or failure to show effectiveness with the treatment delays common in human studies represent some key issues. Nanotechnology, including dendrimers and structural modifications like TPP, discussed earlier, offers excellent potential to increase the efficiency and efficacy of antioxidant therapeutics as their customizable size, stealthy chemistry, and multifunctionality allow them to enhance drug penetration through the BBB. One strategy to improve the delivery of antioxidants to the brain involves the use of the nose-to-brain route, with administration of the antioxidant in specific nasal formulations and its passage to the CNS mainly through the olfactory nerve route [226,227].

Outcomes between individuals following TBI greatly vary, making antioxidant treatment or other treatments for TBI so challenging [228]. The “one-size-fits-all” approach to TBI medicine that has been followed for many years is questionable. Due to this, many researchers have begun to investigate the possibility of using precision medicine techniques to address TBI treatment [228]. TheFDA-approved novel biomarkers for TBI screening, such as GFAP and UCH-L1, which are released from the brain into the bloodstream within 12 h of injury [229]. Notably, personalized stratification based on recently discovered biomarkers can account for individual variability, forming a practical tool that can be used to assist clinical decision making for early TBI diagnosis, and evaluation of therapeutics intervention. This approach holds the potential to overcome the challenges posed by TBI heterogeneity, offering a more tailored and effective strategy for treating TBI patients. An increased understanding of additional biomarkers across the TBI spectrum is needed to improve antioxidant precision medicine in TBI. We stress the importance of further research into this area to improve the clinical efficacy of antioxidant therapy for TBI in the future.

## 7. Holistic Approach to Improve TBI Outcomes

A holistic approach to provide support that looks at the whole person, not just their CNS health, should be taken into consideration for TBI management. TBI alone or in combination with polytraumatic injuries (i.e., TBI + polytrauma) heavily impacts the body, damaging the brain tissue and shifting homeostasis in many bodily systems such as the immune system, GI system, lungs, heart, and gut microbiota [11,12,230]. This systemic insult can result in changes throughout the body that can increase morbidity and even mortality following TBI [231]. Herein, we reviewed a bidirectional relationship between the gut microbiome and the brain, which also plays a role in TBI-associated pathology. Damage to the brain alters the composition of the microbiome; the altered microbiome affects TBI severity, neuroplasticity, and metabolic pathways through various bacterial metabolites [232]. Significant changes in the gut microbiome within two hours following a TBI was demonstrated in rats, and dysbiosis persisted throughout the study period of 7 days [233]. Furthermore, gut dysbiosis was associated with neuronal loss 3 months after TBI [234]. Notably, emerging research indicates a potential link between the gut microbiome and neurological health [232,233]. The interaction of the CNS and gut signaling pathways includes chemical, neural, metabolic, immune, and endocrine routes, and imbalances in these pathways have been associated with neurological disorders like PD, MS, and AD [235]. Therefore, microbiota manipulation has been proposed as a treatment target for such diseases [236]. While this field of research evolves, maintaining a healthy gut through diet and lifestyle may positively impact outcomes following TBI.

The gut–brain axis suggests that a bidirectional communication between the gut and the brain may influence neurological conditions. A balanced microbiome may contribute to antioxidant production, potentially influencing our body’s ability to combat oxidative damage [237]. Recently, it has been shown that the intake of antioxidant compounds might modulate the composition of beneficial microbial species in the gut, and these commensal bacteria often exhibit antioxidant properties [238]. Thus, the antioxidant supplements and balanced microbiome complement each other due to their mutualistic associations. Probiotic-derived metabolites such as butyrate, propionate, and acetate may serve as alternative energy sources for an injured brain and may improve mitochondrial function following TBI [230,239]. Further supporting the benefit of antioxidants, polyphenol antioxidants such as quercetin, resveratrol, and flavonoid intervention have shown to selectively inhibit pathogenic bacteria in the gut [240]. Additionally, short-chain fatty acids (SCFAs), the main metabolites produced in the colon by bacterial fermentation may contribute to host energy production and ROS modulation [241]. Furthermore, the gut microbiota has been shown to regulate key transcriptional co-activators, transcription factors and enzymes involved in mitochondrial biogenesis, such as the PGC-1α, SIRT1, and AMPK genes [241]. Thus, metabolites produced by commensal gut microbiota, including the beneficial SCFAs, might influence key mitochondrial functions related to TBI pathobiology such as energy production, mitochondrial biogenesis, and redox balance, making them a potential therapeutic target.

Due to the high energy demands exist during the repair of an injured brain; and growing our understanding of brain-gut microbiota crosstalks for the host’s overall health, we have briefly highlighted the existence of interactions between the brain, gut microbiota and mitochondrial redox homeostasis. However, the underlying mechanisms through which antioxidants might influence the gut–brain axis to exert neuroprotection in TBI is yet to be fully elucidated. This knowledge gap is of paramount clinical significance.

## 8. Conclusions

Emerging evidence indicates that mitochondrial homeostasis is central to the secondary injury cascade in TBI pathology, which lacks approved therapy. Loss of this homeostasis, including redox imbalance, excitotoxicity, calcium overload, bioenergetics failure, and apoptosis, are the main participants in mitochondria-centered damage following TBI, contributing to neuronal death and long-term neurobehavioral sequelae. Thus, mitochondria-targeted antioxidant strategies in TBI have been increasingly studied, as their maintenance could potentially preserve neuronal homeostasis and crucial brain functions. Properly selecting mitochondria-targeted antioxidants, greater understanding of the underlying injury mechanisms, better-tailored treatments, and the application of novel pharmacological methodology offer new insights into the successful management of TBI, and its translation from bench to bed. Therefore, the antioxidants reviewed here could be a viable therapeutic option to minimize secondary damage and improve the quality of life after TBI. However, further research using antioxidants as a treatment for TBI is necessary in order to move towards adding them into routine care for TBI.

## Figures and Tables

**Table 1:** Natural ROS scavengers.

ROS Scavengers	Properties and Mechanisms of Action
**Enzymatic ROS scavengers**	
Superoxide dismutase (SOD)	Enzyme. Converts superoxide radicals into oxygen and H_2_O_2_.
Catalase (CAT)	Enzyme in the peroxisomes. Neutralizes H_2_O_2_ in water.
Glutathione peroxidase (GPx) Thioredoxin system: Thioredoxin (Trx), Peroxiredoxin (Prx), Thioredoxin reductase (TrxR)	Thiol-dependent enzymatic antioxidants. Neutralize H_2_O_2_ and are recycled by nicotinamide adenine dinucleotide phosphate (NADPH) as a cofactor.
**Non-enzymatic ROS scavengers**	
Vitamin A (retinol) or carotenoids	Fat-soluble antioxidant. Donates electrons to neutralize free radicals.
Vitamin E (tocopherols and tocotrienols)	Fat-soluble antioxidant. Scavenges lipid peroxyl radicals.
Vitamin C (ascorbic acid)	Water-soluble antioxidant. Donates electrons to neutralize free radicals. Scavenge superoxide.
Carotenoids	Found in various fruits and vegetables. Of the ~600 types of carotenoids, some can synthesize Vitamin A. Neutralizers of ROS.
Polyphenols	Ubiquitously present in fruits and vegetables. Free-radical scavenger.
Flavonoids	Phytochemicals present in plants, fruits, and vegetables. Scavengers of ROS.
Pycnogenol (PYC)	Combination of bioflavonoids with robust capacity to scavenge free radicals.
Alliin	Found in both natural and synthetic compounds. A bioactive compound derived from garlic. Superoxide scavenger.
Allicin	Synthesized from alliin. Inhibits superoxide, nitric oxide (NO) and hydroxyl radicals.
Minerals (copper, zinc and selenium, magnesium)	Precursors to antioxidants that help regulate free radicals.
Coenzyme Q10 (CoQ10), coenzyme Q (CoQ)	Lipid antioxidant. Essential component of the ETC. Protects cells from oxidative damage.
Glutathione	Tripeptide. Detoxifies ROS. Maintains redox balance.
NADPH	NADPH, as a cofactor independently and with redoxins, plays a crucial role in ROS detoxification.
Cytochrome C	Endogenous heme protein located in mitochondria. Oxidized cytochrome C is able to scavenge superoxide radicals.

**Table 2:** Synthetic ROS scavengers and detoxifiers.

ROS Scavengers and Detoxifiers	Properties and Mechanisms of Action
**Non-targeted compounds**	
MnTBAP	O_2_^•−^ scavenger. Possesses SOD- and catalase-like activity. Also scavenges ONOO^−^.
Cysteine	Amino acid. O_2_^•−^ scavenger.
Tiron	Reduced and oxidized Tiron species. Reacts with O_2_^•−^ radical.
Carboxy-PTIO	Specific NO scavenger. Reacts with O_2_^•−^ radical.
Phenelzine	FDA-approved drug. MAO inhibitor. Aldehyde-scavenging properties partially protect against oxidative damage.
**Mitochondria-targeted compounds**	
MitoVit-E	Vitamin E attached to TPP. Reduces mitochondrial oxidative damage.
MitoQ	CoQ10 derivative linked with TPP. Scavenges mitochondrial ROS.
Plastoquinone (SkQ1)	Targeted antioxidant. Scavenges mitochondrial ROS.
Edaravone	Used clinically as a neuroprotective compound. Reduces oxidative damage and lipid peroxidation.
Mito TEMPOL	Cell permeable, stable nitroxide. SOD mimetic.
Elamipretide (SS-31)	Cationic tetrapeptide freely permeable to the mitochondria. Reduces the production of toxic ROS.
Cerium oxide nanoparticles (Nano-CeO2)	Cerium atoms linked by oxygen atoms. Scavengers of ROS.
Metalloporphyrins	Manganese and iron complexes. Synthetic catalytic antioxidants that mimic the body’s own antioxidant enzymes.
Phenyl-tert-butylnitrone (PBN)	Nitroxide radical. ROS-scavenging properties.
**Glutathione precursors**	
NAC	A cysteine prodrug. Replenishes intracellular glutathione level.
NACA	N-acetyl cysteine (NAC) analog.Glutathione precursor.
D-NAC	Dendrimer-tagged NAC. Serves as a prodrug to synthesize glutathione.
S-adenosyl methionine (SAMe)	SAMe is processed stepwise into cysteine synthesis, and ultimately synthesize glutathione.

**Table 3:** Signaling pathway modulators.

Pathway Modulators	Properties and Mechanisms of Action
**Nrf2 activators**	
Omaveloxolone (RTA-408)	Synthetic compound. FDA-approved for the treatment of FA. Prevents Nrf2 degradation.
Dimethyl fumarate (DMF)	Synthetic compound. Activates the Nrf2 pathway and AKT pathway.
Curcumin	Derived from turmeric. Activates the Nrf2 pathway.
Sulforaphane	Naturally found in cruciferous vegetables. Activates Nrf2 by inhibiting Keap1.
Epigallocatechin gallate (EGCG)	Abundant in green tea. Activates the Nrf2 pathway and has antioxidant and anti-inflammatory properties.
Quercetin	Present in various fruits, vegetables and grains. Activates Nrf2 and SIRT1.
Oltipraz	Synthetic compound. Activates Nrf2 by modifying Keap1.
Bardoxolone methyl	Synthetic compound. Activates the Nrf2 pathway.
**SIRT1, PGC-1α, and mTOR modulators**	
Resveratrol	Natural polyphenol compound. Most-relevant SIRT1 and mTOR modulator, AKT activator, Nrf2 activator and PGC-1α activator.
Naringenin	Natural citrus flavonoid. Modulates SIRT1.
SRT2104	Synthetic compound. SIRT1 activator.
1,4-dihydropyridine derivative	Synthetic compound. SIRT1 activator.
Naphthofuran derivative	Synthetic compound. SIRT1 activator.
Bisarylaniline derivative	New synthetic analog. SIRT1 activator.
Berberine	Small molecule isolated from various plants, mainly used in Chinese traditional medicine. PGC-1α activator.
Metformin	Anti-diabetic drug. Activator of AMPK, which further regulates PGC-1α.
Rapamycin/Sirolimus	Bacterial origin natural product. mTOR inhibitor and increases antioxidant defense.
Everolimus	Newly developed mTOR inhibitor. Rapamycin analog.
Temsirolimus	Newly developed mTOR inhibitor. Rapamycin analog.
